# Association between foot types defined by static and dynamic measures, and the centre of pressure during gait

**DOI:** 10.1186/1757-1146-7-S1-A55

**Published:** 2014-04-08

**Authors:** Su Liao, Hannah L Javis, Anmin Liu, Christopher J Nester, Peter P Bowden, Richard K Jones, Kaiyu Xiong

**Affiliations:** 1 Sport Science College, Beijing Sport University, Beijing, 100084, China; 2 School of Health Sciences, University of Salford, Salford, M6 6PU, UK

## Background

Foot types (e.g. pronated, supinated foot) are used for clinical reasoning [1] and widely assumed to be related to centre of pressure (COP) patterns [2,3]. Specifically, a pronated foot will demonstrate a medially deviated COP. It follows that COP could be a measure of foot type and inferences about function extrapolated from it. The purpose of this study was to investigate whether COP parameters differ between foot types.

## Methods

**Figure 1:**
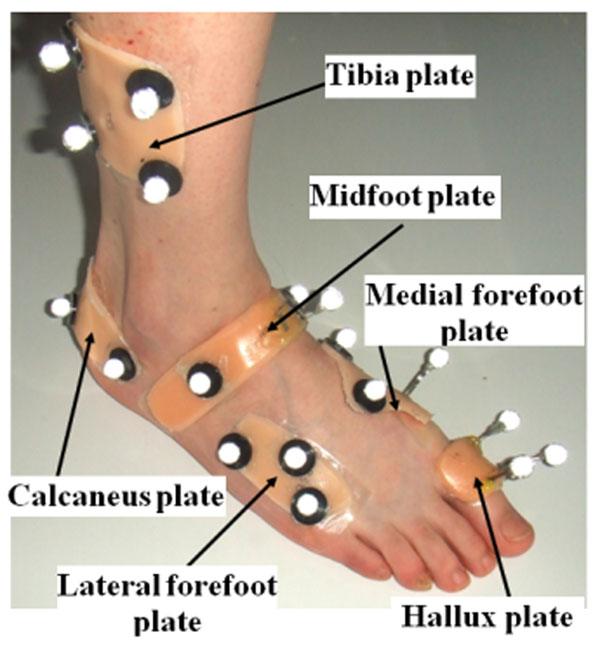
Five segment foot kinematic model.

Static foot posture, foot kinematics and COP data were collected on 90 healthy subjects during walking (Figure 1). The subjects were classified as pronated, supinated, and neutral groups using three static and four dynamic methods (table 1). COP lateral and medial excursion area, COP lateral medial difference (COP_LMD), and COP index (COP_I) were calculated for different phases of stance [4-6]. Independent T test and correlations were calculated among the different groups.

**Table 1:** 

Classification method	Pronators	Supinators
Foot Posture Index(FPI)	≥7	≤-1

Resting Calcaneal Stance Position (RCSP)	≤-2°	≥3°

Difference between NCSP^*^ and RCSP	≥8°	≤4°

Peak Rearfoot Eversion(PRE)	≤-6.1°	≥-1.1°

Time of Peak Rear foot Eversion (TPRE)	≥38%	≤26%

Range of Rearfoot Eversion (RRE)	≥16.3°	≤10.5°

Maximum Mid Foot Dorsiflexion	≥6.4°	≤1.1°

*NCSP:neutral calcaneal stance position

## Results

*Pronated feet* (based on FPI) demonstrated more medial excursion of the COP from heel strike to heel off (p<0.05). Pronated feet classified by NCSP-RCSP demonstrated higher COP_I during HO-TO (p<0.05).

*Supinated feet* classified by NCSP-RCSP and RRE had more medial excursion of the COP (COP-ME) during HO-TO (p<0.05). Feet classified as supinated by TPRE resulted in a greater COP-LMD in a stance (p<0.05) and their COP_I was statistically significantly higher. Feet classified as supinated by RRE showed higher COP-LMD value during HO-TO (p<0.05). The statistical results showed a weak relationship between COP parameters of different foot types (r<0.27). Dynamic measures of foot type showed a slightly stringer association to COP measures than static measures of foot type.

## Conclusion

Over all, whilst there were some differences between foot types in some COP measures, the meaning of the observed differences does not support the hypothesis that COP parameters are strongly indicative of specific foot types. Thus, COP measures should not be used to infer foot kinematic nor foot function.
